# Oral co-administration of a bacterial protease inhibitor in the vaccine formulation increases antigen delivery at the intestinal epithelial barrier

**DOI:** 10.1016/j.jconrel.2018.11.025

**Published:** 2019-01-10

**Authors:** Lorena M. Coria, Gabriela S. Risso, Francisco F. Guaimas, Mariana C. Ferrero, Laura Bruno, Karina A. Pasquevich, Juliana Cassataro

**Affiliations:** a Instituto de Investigaciones Biotecnológicas (UNSAM-CONICET), Universidad Nacional de San Martín, Buenos Aires, Argentina; b Instituto de Estudios de la Inmunidad Humoral (CONICET-UBA), Facultad de Farmacia y Bioquímica, Universidad de Buenos Aires, Buenos Aires, Argentina

**Keywords:** Bacterial protease inhibitor, Intestinal barrier, Vaccine adjuvant, Enterocytes, Oral delivery

## Abstract

The study of capture and processing of antigens (Ags) by intestinal epithelial cells is very important for development of new oral administration systems. Efficient oral Ag delivery systems must resist enzymatic degradation by gastric and intestinal proteases and deliver the Ag across biological barriers. The recombinant unlipidated outer membrane protein from *Brucella* spp. (U-Omp19) is a protease inhibitor with immunostimulatory properties used as adjuvant in oral vaccine formulations. In the present work we further characterized its mechanism of action and studied the interaction and effect of U-Omp19 on the intestinal epithelium. We found that U-Omp19 inhibited protease activity from murine intestinal brush-border membranes and cysteine proteases from human intestinal epithelial cells (IECs) promoting co-administered Ag accumulation within lysosomal compartments of IECs. In addition, we have shown that co-administration of U-Omp19 facilitated the transcellular passage of Ag through epithelial cell monolayers *in vitro* and *in vivo* while did not affect epithelial cell barrier permeability. Finally, oral co-delivery of U-Omp19 in mice induced the production of Ag-specific IgA in feces and the increment of CD103^+^ CD11b^−^ CD8α^+^ dendritic cells subset at Peyer's patches. Taken together, these data describe a new mechanism of action of a mucosal adjuvant and support the use of this rationale/strategy in new oral delivery systems for vaccines.

## Highlights

• The bacterial protease inhibitor U-Omp19 limits antigens proteolysis by enterocytes.
• Oral co-administration of U-Omp19 increases antigen half-life inside enterocytes.
• U-Omp19 oral administration does not affect epithelial cell barrier permeability.
• Oral co-delivery of U-Omp19 increases frequency of dendritic cells bearing antigen.
• U-Omp19 increases the half-life and immunogenicity of cholera toxin subunit B antigen.

## Graphical abstract

U-Omp19 protease inhibitor protects oral co-delivered antigen digestion by inhibiting intestinal lumen proteases (1) and brush border and lysosomal proteases from enterocytes (2) while inducing recruitment of dendritic cells and increasing the frequency of antigen bearing dendritic cells at immune inductive sites (3).

**Figure:**
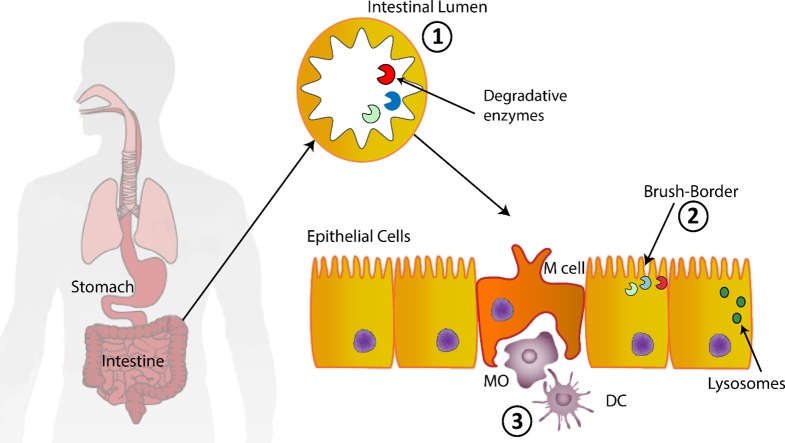

## 1 Introduction

There is no vaccine that is more applicable to mass immunization in field settings than oral vaccine. The lack of need for needles and syringes allows untrained personnel to carry out immunization and avoids major safety issues. Additionally, it has less stringent criteria for purity of vaccines. Oral administration of medicines is a general and historical medical practice and is generally accepted by people of diverse cultures. Yet, there is a relative paucity of oral vaccines in current medical practice. Most oral vaccines available today are either attenuated or killed microorganisms that can survive intestinal degradation either by replicating in the gut or by virtue of having digestion-resistant bacterial walls [1]. Attenuated or live vaccines will always be associated with safety concerns. For example, several adverse reactions associated with oral polio or rotavirus vaccines have been described [2].

Although oral vaccination using peptide or protein antigens (Ags) may not have severe adverse effects, they face great obstacles such as (i) low gastric pH and proteolytic digestion by gastrointestinal (GI) enzymes, (ii) the intestinal epithelial barrier that hampers efficient absorption and transport of intact Ag to the gut associated lymphoid tissues (GALT) and (iii) induction of immune tolerance. These problems explain the general immune hyporresponsiveness and high doses needed for oral vaccines [3].

It is important to state that protein degradation at the GI tract not only occurs at the lumen by stomach and pancreatic proteases. The surface area of the absorbing cells (enterocytes) of the small intestine is considerably increased by the surface membrane being folded into microvilli at the apical pole of the enterocyte. This membrane structure is known as the brush border membrane (BBM) and is rich in hydrolytic enzymes, mainly peptidases. These are at least 12 in number and comprise endopeptidases and both amino- and carboxy-exopeptidases. The intestinal brush border constitutes a functional organelle subserving terminal digestion and absorption of the end products of ingested food [4,5].

After oral ingestion, macromolecules that escape digestion by pancreatic and brush border enzymes are likely to be taken up into epithelial cells by endocytosis. During transcytosis to the lamina propria, macromolecules can be transported through non-selective fluid-phase pinocytosis or through selective receptor-mediated endocytosis. The initial process of both mechanisms involves membrane invagination with formation of clathrin-coated pits at the base of the invagination. In the non-selective fluid-phase transport macromolecules are mostly directed toward the degradative pathway where they enter in endocytic vesicles that then fuse with lysosomes and are subjected to intracellular hydrolysis by lysosomal enzymes. This process normally mediates the uptake of nutrients and soluble protein Ags by enterocytes [6].

Thus, after bypassing stomach and pancreatic proteases, the Ag faces proteolytic digestion by brush border and intracellular proteases at enterocytes to cross the intestinal epithelial barrier and reach antigen presenting cells (APCs) at immune inductive sites. In this context, development of an appropriate Ag delivery system is crucial to protect the Ag from enzymatic degradation by gastric and intestinal proteases and to deliver the Ag across the epithelial barrier. Moreover, the formulation should maintain the capacity to elicit potent and long-lasting antibody and cellular immune responses.

U-Omp19 is a protease inhibitor from *Brucella* spp. that has been studied as a mucosal vaccine adjuvant. We have shown that it can bypass the harsh environment of the gastrointestinal tract by inhibiting proteases present at the stomach and intestine lumen limiting co-administered Ag digestion and consequently increasing Ag amount at immune inductive sites [7]. Moreover, U-Omp19 partially inhibits Ag digestion by lysosomal proteases inside dendritic cells (DCs) increasing Ag half-life and enhancing Ag presentation to T cells [8] and promoting mucosal and systemic Ag-specific immune responses (Th1, Th17 and CD8^+^ T cells) after co-administration with the Ag [[7], [8], [9], [10]]. In this work, we investigate U-Omp19 effect on luminal Ag transport through the intestinal epithelial cell barrier.

## 2 Materials and methods

### 2.1 Ethics statement

All experimental protocols of this study were conducted in agreement with international ethical standards for animal experimentation (Helsinki Declaration and its amendments, Amsterdam Protocol of welfare and animal protection and National Institutes of Health guidelines, NIH, USA). The protocols of this study were approved by the Institutional Committee for the Care and Use of Animals for experimentation from the University of San Martin (UNSAM). CICUAE N° 5/2014.

### 2.2 Ags and adjuvants

Chicken egg OVA grade V (Sigma-Aldrich) and cholera toxin subunit B (Sigma-Aldrich) were used as Ags. Recombinant U-Omp19 was obtained as previously described [11]. LPS contamination from U-Omp19 was adsorbed with sepharose–polymyxin B (Sigma Aldrich). Endotoxin determination was performed with a Limulus amoebocyte chromogenic assay (Lonza). All U-Omp19 preparations used contain <0.1 endotoxin units per milligram of protein. Control inhibitor Leupeptin was purchased from Sigma-Aldrich.

### 2.3 Mice and immunizations

Eight-week-old female BALB/c mice were obtained from University of San Martin (UNSAM). Housing was performed at the animal resource facility of UNSAM. Mice were fasted 2 h prior and after oral immunizations.

### 2.4 Cells

Caco-2 cells were maintained in RPMI 1640 medium supplemented with 10% heat-inactivated fetal bovine serum, 1 mM sodium pyruvate, 2 mM l-glutamine, 100 U/ml penicillin, 100 μg/ml streptomycin, at pH 7.4 in a humidified atmosphere (5% CO_2_, 37 °C). The cells were grown under standard conditions until 60–70% confluency. Cells from passages 10–30 were used in all the experiments. HT29 cells were maintained in DMEM: ham F12 medium (1:1 mixture) supplemented with 10% heat-inactivated fetal bovine serum, 1 mM sodium pyruvate, 2 mM l-glutamine, 100 U/ml penicillin, 100 μg/ml streptomycin.

### 2.5 Determination of BBMs proteolytic activity

Brush-border membrane vesicles were prepared by MgCl_2_ precipitation [12]. Briefly, small intestine mucosa was scrapped using a hypotonic solution to obtain disrupted cells. Then, a solution containing 100 mM MgCl_2_ was added to the homogenate and fractionation by centrifugation was performed. Vesicles containing brush-border membranes were incubated alone or with different concentrations of U-Omp19 for 1 h at room temperature and then 1 mg/ml substrate (casein–BODIPY FL, Molecular Probes) was added. Fluorescence emission was measured with a fluorescence plate reader (FilterMax F5, Molecular Devices).

### 2.6 Evaluation of Ag internalization and degradation by flow cytometry

Caco-2 cells (2 × 10^6^ cells) were seeded in 24-well plates and incubated with the Ag: OVA-FITC (100 μg/ml), DQ-OVA (50 μg/ml), Casein-FITC (100 μg/ml), Casein-BODIPY (25 μg/ml), CTB-Alexa Fluor 647 (10 μg/ml), dextran-FITC (10 μg/ml) or Yellow green particles (ratio 1 cell: 1000 particles) alone or with the protease inhibitor (U-Omp19, 100 μg/ml) during 3 h. Then, cells were washed and Ag internalization and/or degradation was measured by flow cytometry (Partec Cytometer) and further analyzed using FlowJo 10× software (TreeStar Inc).

### 2.7 Cathepsin L inhibition

Caco-2 or HT29 cells were incubated with recombinant purified U-Omp19 at different concentrations (10, 25, 50 and 100 μg/ml) or Leupeptin (10 μg/ml) for 3 h at 37 °C, 5% CO_2_. Proteolytic activity was then analyzed by adding 50 μM of cathepsin L specific fluorogenic substrate (CBZ-Phe-Arg)_2_ (Invitrogen) for 30 min at 37 °C, 5% CO_2_. Fluorescence emission was measured for 4 h (100 cycles) using a microplate reader (Filtermax F5, Molecular Devices) with 485 nm (excitation) and 535 nm (emission) filters and Multimode detection software. Data were analyzed using Prism 6.0 (GraphPad Software).

### 2.8 In vitro protease inhibition assay

Caco-2 cells (2 × 10^6^ cells) were seeded in chamber slides (Nunc, Lab-tek, ThermoFisher) and after 15 days of culture incubated with recombinant purified U-Omp19 at different concentrations (50 or 100 μg/ml) or Leupeptin (10 μg/ml) for 3 h at 37 °C, 5% CO_2_. Then, 50 μM of cathepsin L specific fluorogenic substrate (CBZ-Phe-Arg)_2_ and 50 nM Lysotracker dye (Invitrogen) were added in HBSS medium for 30 min at 37 °C, 5% CO_2_. Proteolytic activity of cathepsin L was evaluated by confocal microscopy in living cells. Each chamber was evaluated in at least five random fields, the microscope used was an IX-81 attached with a FV-1000 confocal module (Olympus Corp.) and an Objective 60× PlanApo (Olympus Corp.). Colocalization analysis was made with ImageJ (NIH, US) and ICY Image Software (Institute Pasteur, France). ImageJ Plugging using a Rolling Ball of 50 pixels was used to subtract background of each image. Measurements of the maximum and minimum value of Threshold for each image were performed using ImageJ. The ICY Image Software was used to set the ROIs using the Maximum and Minimum values and then Manders coefficient was calculated for each image.

### 2.9 Ag internalization in vitro

Caco-2 cells were incubated in glass cover slips for 3 h with OVA Alexa Fluor 647 (50 μg/ml) or CTB Alexa Fluor 647 (10 μg/ml) alone or in presence of different concentrations of U-Omp19 (25 and 50 μg/ml) or Leupeptin (10 μg/ml) in medium. Then, cells were washed, fixed with 2% paraformaldehyde, permeabilized with 0.2% saponin and stained with mouse anti–human Lamp-2 (1:500; BD Biosciences). Secondary antibody anti-mouse IgG Alexa Fluor 546 (Invitrogen) was used. Then, slides were mounted with Aqua-Poly/Mount (Polysciences) and analyzed using an IX-81 microscope attached with a FV-1000 confocal module. For quantification of colocalization Lamp-2/OVA or Lamp-2/CTB, the Manders plugin of ICY Image Software was used and the coefficient was calculated. Single ROIs (Lamp-2^+^ compartments) were determined automatically with Spot Detector plugin detecting bright spots in dark background using a scale and sensitivity of 7 pixels and size filtering of 50–50,000 pixels. Manders colocalization coefficient was measured in each ROI using Manders plugin.

### 2.10 Transport studies using transwell filters

Caco-2 cells were seeded in the upper chamber of tissue culture polycarbonate membrane filters (Transwell®, pore size 0.4 μm, 6.5 mm inner diameter; Corning NY) in 24-well plate at a seeding density of 1 × 10^5^ cells/cm2. The culture medium was added to both the donor and the acceptor compartment. Medium was changed every second day. The cells were left to differentiate for 19–21 days after seeding with monitoring of TEER values.

During the experiments measurement of TEER was performed to evaluate possible damage of the cellular monolayer. The values of TEER were determined by measuring the potential difference between the two sides of the cell monolayer using a (Millicell ERS-2, Millipore Corporation, Billerica, MA) connected to a pair of chopstick electrodes. On the day of experiments, the cells were washed twice with phosphate buffered saline (PBS) and pre-equilibrated for 1 h with Hank balanced salt solution (HBSS) buffered at pH 7.4. After removing the medium, Caco-2 cell monolayers were treated with OVA alone (0.5 mg/ml) or plus U-Omp19 (12.5, 25 or 50 μg/ml) or Leupeptin (10 μg/ml) solutions in HBSS at pH 7.4 at apical compartment for 3 h and the basolateral medium used was HBSS pH 7.4. Samples (100 μl) were taken every hour from the basolateral side and the fluorescence measured in a microplate fluorescence reader (Filtermax F5, Molecular Devices). The excitation and emission wavelengths were 590 and 670 nm, respectively.

The TEER was measured after the treatment and then the cells were washed with HBSS and lysed with lysis buffer (Tris 0.02 M pH 8, NaCl 0.15 M, NaF 0.1 M, PMSF 1 mM, Na_3_VO_4_ 1 mM, 10% glycerol, 1% NP-40, leupeptin and aprotinin). Also, the passage of the enzyme horseradish peroxidase (HRP) was used to evaluate monolayer integrity using a 3,3′,5,5′-Tetramethylbenzidine (TMB) solution as substrate to detect HRP presence at the basolateral medium.

### 2.11 Western blot

Amounts of OVA transported across epithelial monolayers were analyzed by western blot in cell lysates and in basolateral medium (previously precipitated with TCA). In lysates, calnexin levels were used as loading control. Samples were run in a SDS-PAGE and then transferred to a nitrocellulose membrane (Amersham) and then blocked with TBS-Tween 0.05% ON at 4 °C. Chicken egg ovalbumin was detected at ~45 kDa after probing with IgG1 monoclonal anti-OVA (Thermofisher) at a dilution of 1:2000. Calnexin was detected with polyclonal anti-calnexin antibodies at a dilution 1:500 overnight at 4 °C on a rocking platform. An IRDye 680 and 800 nm labeled anti-mouse IgG were used as secondary antibodies at a dilution of 1:20.000. Fluorescence was detected using Odissey infrared imaging System (LI-COR Biosciences). Image Studio 5.2 software was used to quantify Mean ± SEM relative density of the bands normalized to calnexin or to total OVA signal.

### 2.12 Intestinal tissue immunofluorescence

BALB/c mice were orally administered with OVA Alexa Fluor 647 (100 μg) alone or OVA Alexa Fluor 647 plus U-Omp19 (150 μg) and 2 h later intestine sections were excised (duodenum, jejunum and ileum) and prepared for immunofluorescence. Intestine sections were fixed with 4% paraformaldehyde overnight and then immersed in 30% sucrose buffer overnight. Then tissue was embedded in optimum cutting temperature compound (OCT, Biopack) and frozen at −80 °C for cryosectioning.

Cryostat sections (~10 μm) were mounted on positive charged glass slides (Biogenex), washed with PBS and blocked with 1% BSA, 5% horse serum diluted in PBS for 1 h at room temperature. Epithelial cell glycoproteins were stained using Alexa Fluor 555 conjugated wheat germ agglutinin (WGA) (ThermoFisher) and M cells were detected using Fluorescein labeled *Ulex europaeus* Agglutinin I (UEA I, Vector Labs) both staining were performed in 1% BSA, 5% horse serum diluted in PBS for 30 min at room temperature. In other slides, rabbit anti-U-Omp19 polyclonal Ab (1:1000) or mouse anti-CD11c mAb (1:500) were used as primary antibodies and anti-rabbit Alexa Fluor 488 or anti-mouse Alexa Fluor 546 as secondary antibodies. After extensively washing in PBS, Sections were mounted using FluorSave reagent (Calbiochem). Slides were then analyzed with IX81 microscope with a Confocal module attached model FV1000 using a 40× NA 1.3 oil immersion objective and a 10× NA 0.30 objective. The images were taken in 4096 × 4096 pixels in all fields and each channel token and a Region Of Interest (ROI) was selected and cropped. Finally, the background was subtracted in the three channels cropped and added to obtain the final image.

### 2.13 Determination of specific Ab responses

BALB/c mice (*n* = 5/group) were orally immunized on days 1, 2, 3, 8, 9 and 10 with saline, CTB (10 μg) or CTB plus U-Omp19 (150 μg). Fecal extracts from immunized mice were prepared as described previously [10]. CTB-specific IgA in fecal extracts and IgG and the isotypes (IgG1 and IgG2a) in serum were determined by ELISA as described previously [7].

### 2.14 Dendritic cell subset characterization by flow cytometry

Mice were administered orally with OVA (100 μg) alone or plus U-Omp19 (150 μg) and 18 h later Peyer's patches (PPs) cell suspensions were obtained as described previously [7]. Before staining, cells were incubated for 15 min at 4 °C with TruStain fcX (anti-mouse CD16/32, Biolegend). Then, cells were stained with fluorochrome conjugated antibodies: anti-CD11c (HL3), anti-CD11b (M1/70), anti-CD8α (53–6.7), anti-MHC- II (M5/114.15.2) and anti-CD103 (M290) for 30 min at 4 °C. Afterwards cells were washed and fixed with paraformaldehyde for flow cytometry analysis. Monoclonal Abs were purchased from eBiosciences (San Diego, CA), BioLegend (San Diego, CA) and BD Biosciences (Franklin Lakes, NJ). Samples were acquired for 5 colour analysis using a FACS Aria II flow cytometer (BD Biosciences) with DIVA software (BD Biosciences) and further analyzed using FlowJo 10× software (TreeStar Inc).

#### 2.14.1 Ag internalization in vivo

BALB/c mice were immunized with OVA Alexa Fluor 647 (100 μg) or OVA AF647 plus U-Omp19 (150 μg) and 4 h later MLNs were excised, and cells suspensions obtained. Then, cells were stained with fluorochrome conjugated antibodies: anti-CD11c (HL3), anti-CD11b (M1/70), anti-CD8α (53–6.7), anti-MHCII (M5/114.15.2) and anti-CD103 (M290) for 30 min at 4 °C. Afterwards cells were washed and fixed with paraformaldehyde for flow cytometry analysis.

### 2.15 Statistical analysis

GraphPad Prism 6 software (GraphPad, San Diego, CA) was used for Statistical analysis and plotting. One-way ANOVA test followed by the Bonferroni multiple-comparison post-test was used to analyze densitometry, OVA fluorescence using transwell filters experiments and Manders colocalization coefficient results. The antibody titers and Flow cytometry data were compared using the nonparametric Mann-Whitney *U* test.

## 3 Results

### 3.1 U-Omp19 reduces the degradative capacity of brush border and intracellular proteases from intestinal epithelial cells

**Fig. 1:**
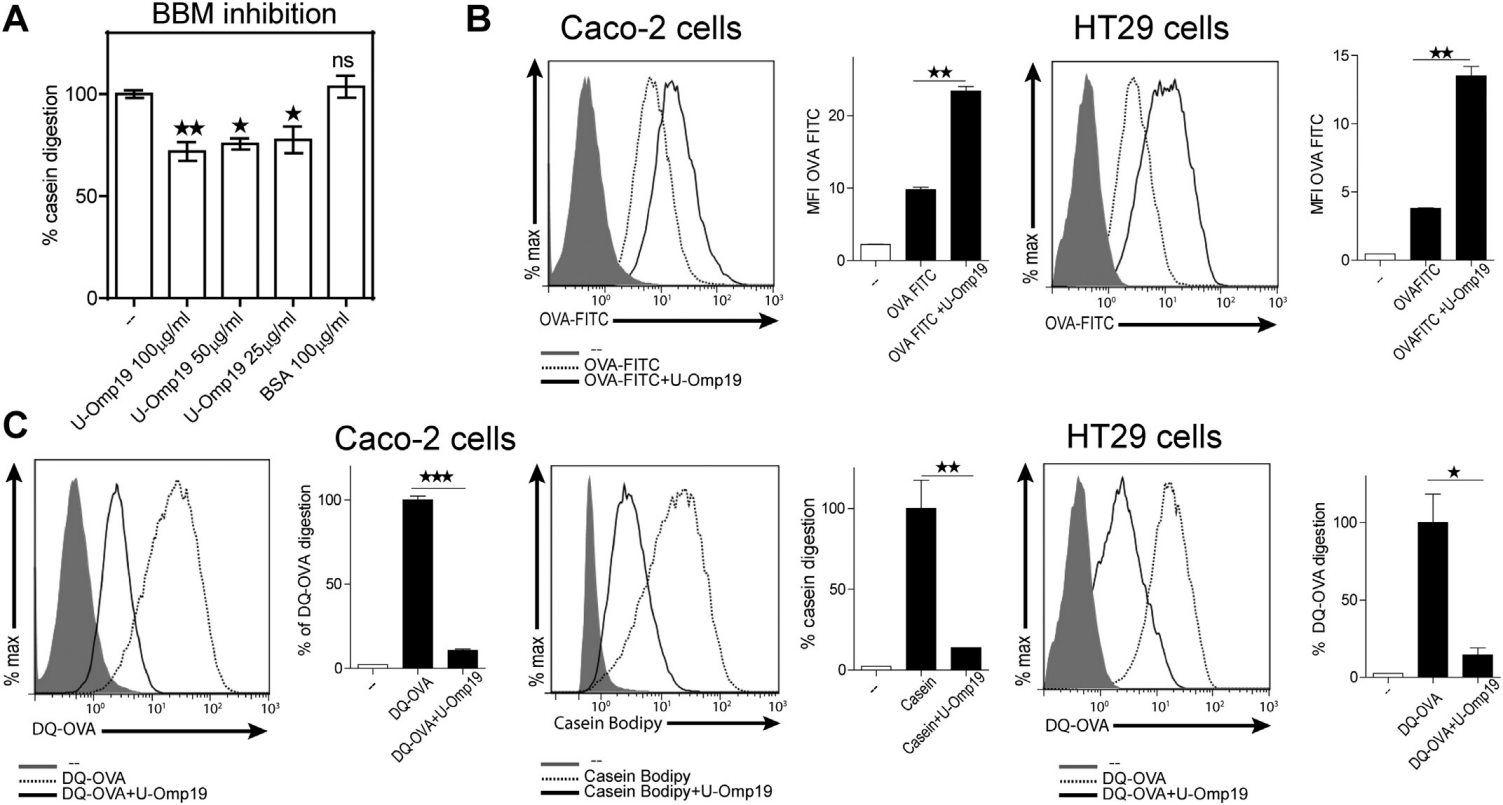
U-Omp19 partially inhibits brush border and intracellular proteases from enterocytes. A. Brush-border membranes (50 μg) proteolytic activity was determined after incubation for 1 h with different amounts of U-Omp19 (25, 50 and 100 μg/ml) by adding Casein-BODIPY substrate. BSA was used as negative control. Data are presented as percentage of casein digestion ± SEM. *p < 0.05; **p < 0.01; ***p < 0.001 *vs.* no protease inhibitor (--). ANOVA followed by Bonferroni multiple comparison test B. U-Omp19 increases Ag amount inside human intestinal epithelial cells. Caco-2 and HT29 epithelial cell lines were incubated with OVA-FITC alone or plus U-Omp19 during 3 h and then MFI was determined by flow cytometry. Data are presented as histograms and bar graphs ± SEM. C. U-Omp19 inhibits Ag digestion inside human epithelial cells. Caco-2 and HT29 epithelial cell lines were incubated with DQ-OVA or Casein-BODIPY alone or plus U-Omp19 during 3 h and the fluorescence measured by flow cytometry. Results are presented as histograms and bar graphs (mean fluorescence intensity -MFI- ± SEM) for each group. *p < 0.05; **p < 0.01; ***p < 0.001 *vs.* Ag alone. (OVA-FITC, DQ-OVA or Casein-BODIPY). *t*-test.

Digestive enzymes of the small intestinal brush border of enterocytes are responsible for the final stage of luminal digestion prior to absorption [13,14]. Thus, to evaluate protease inhibitor activity of U-Omp19 on brush border proteases, brush border membrane vesicles (BBM) were obtained by the method of Kessler [12]. Different amounts of U-Omp19 (25, 50 and 100 μg/ml) were incubated with BBM and then the protease activity evaluated using a fluorescent substrate (Casein-BODIPY). As shown in Fig. 1A, digestive activity of mouse intestinal BBM was reduced by previous incubation with U-Omp19 (25–100 μg/ml) but not after incubation with BSA (100 μg/ml). Partial inhibition of BBM degradative capacity by U-Omp19 could complement the previously observed inhibition of Ag digestion by pancreatic proteases in the last steps of intestinal digestion and contribute to increasing the amount of undigested Ag reaching inductive sites after oral vaccine delivery.

Then, we assessed if the interaction of U-Omp19 with epithelial cells could increase Ag half-life inside enterocytes. OVA-FITC was used to study Ag internalization while quenched proteins (DQ-OVA and Casein-BODIPY) were used to evaluate Ag digestion after co-delivery with U-Omp19 in Caco-2 and HT29 human cell lines. Analysis of cells by flow cytometry has shown that the amount of intracellular Ag (OVA-FITC) was increased if the Ag was co-incubated with U-Omp19 (Fig. 1B). In contrast, there were no differences in the amount of internalized non-protein Ags (Yellow Green microspheres, dextran FITC. Supplemental Fig. 1). These results indicated that the higher amount of intracellular Ag (OVA-FITC) observed after U-Omp19 co-delivery is not due to an increased endocytic capacity of enterocytes. Moreover, degradation of DQ-OVA and casein-BODIPY was significantly reduced in Caco-2 and HT29 cells if the quenched proteins were co-incubated with U-Omp19 (Fig. 1C). Together these results demonstrate that there is a higher amount of less digested Ag inside enterocytes when it is co-administered with U-Omp19 and this effect is not related to an enhancement of Ag uptake.

### 3.2 U-Omp19 inhibits cathepsin L activity inside acidic compartments of enterocytes increasing the intracellular Ag amount

**Fig. 2:**
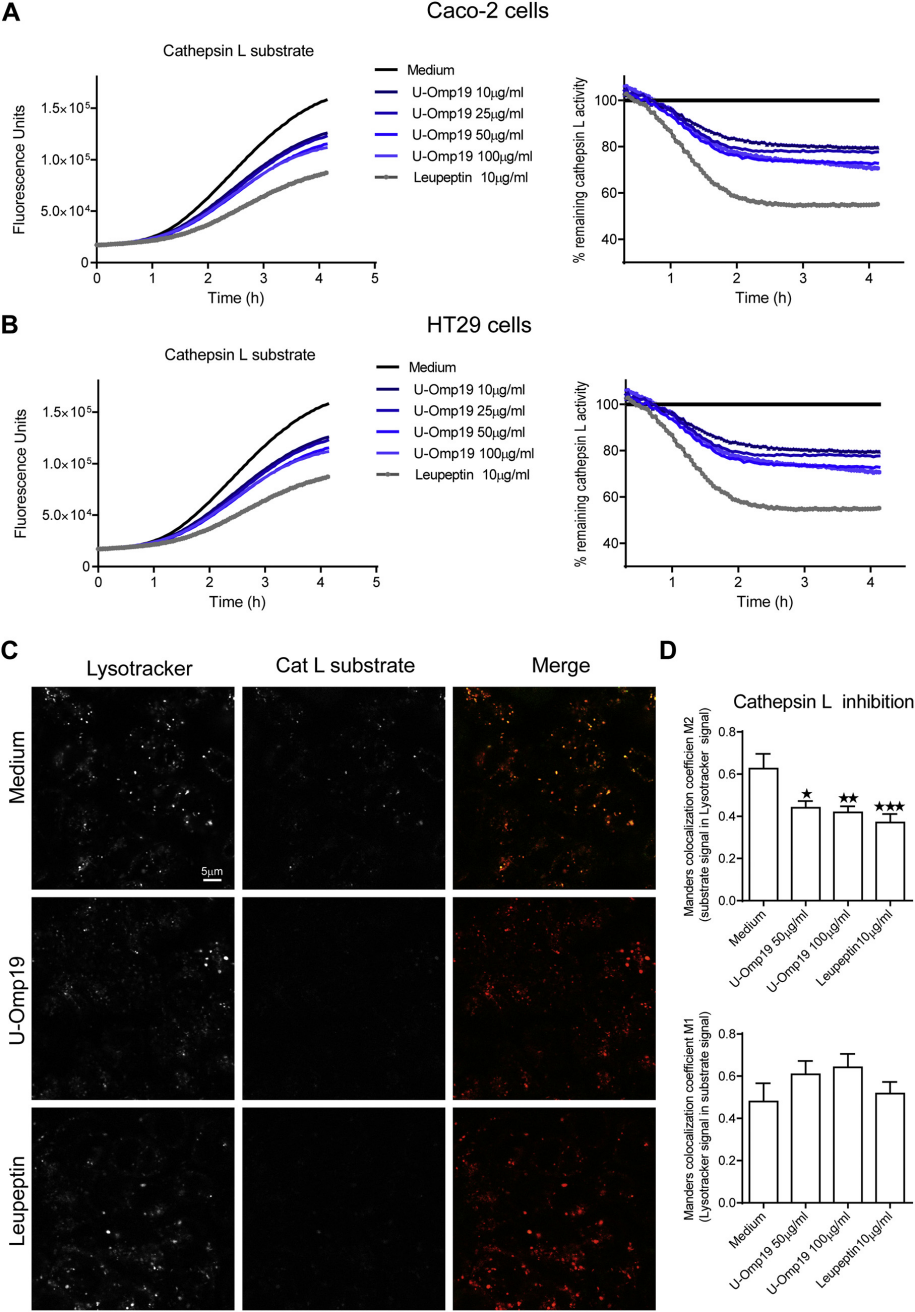
U-Omp19 inhibits cathepsin L activity in human intestinal epithelial cells. Caco-2 (A) and HT29 (B) cells were incubated during 1 h with different concentrations of U-Omp19 or Leupeptin. Then, a specific fluorescence quenched substrate for cathepsin L was added and the reaction was measured in a microplate fluorescence reader for 4 h. Data are shown as arbitrary fluorescence units or percentage of remaining cathepsin L activity over time. C. Confocal microscopy analysis of Caco-2 cells after incubation with the specific quenched substrate for cathepsin L (green) and Lysotracker (red) in presence of medium, U-Omp19 or Leupeptin. Images are representative of most cells examined by confocal microscopy. Colocalization was analyzed by Manders colocalization coefficient (M1 and M2). Data are means of Manders coefficient ± SEM. Scale bars 5 μm. *p < 0.05; **p < 0.01; ***p < 0.001 *vs.* medium. ANOVA followed by Bonferroni multiple comparison test. (For interpretation of the references to colour in this figure legend, the reader is referred to the web version of this article.)

To determine the protease inhibitor activity of U-Omp19 inside living epithelial cells, a cell permeable specific quenched substrate for cathepsin L was used. Caco-2 or HT29 intestinal cells were incubated with different doses of U-Omp19 or with a known cysteine protease inhibitor, leupeptin, for 3 h and then pulsed with cathepsin L specific fluorogenic substrate (CBZ-Phe-Arg)_2_ which fluoresces upon digestion. Live cells incubated with U-Omp19 or leupeptin showed a partial reduction in cathepsin L activity inside both Caco-2 and HT29 cell lines over time (Fig. 2A and B) revealing the capacity of U-Omp19 to inhibit human cathepsin L inside enterocytes.

U-Omp19's ability to inhibit the protease activity of cathepsin L inside live cells was also evaluated by confocal microscopy. Caco-2 cells were incubated with U-Omp19 or leupeptin for 3 h and then washed and labeled with Lysotracker Red DND-99 dye. Lysotracker is a cell-permeant dye, which accumulates and fluoresces in acidic compartments in live cells. Simultaneously with Lysotracker, cells were incubated with the quenched fluorogenic substrate of cathepsin L for 30 min. Co-localization of the digested substrate (fluorescent green) and Lysotracker (red) was evaluated by Manders Coefficient in living cells. Results indicated that U-Omp19 and leupeptin reduced significantly the co-localization of Lysotracker (lysosomes) with the digested cathepsin L substrate compared with the incubation of Caco-2 cells with medium alone (Fig. 2C and D). These results confirmed U-Omp19's inhibition of lysosomal cathepsin L activity inside live Caco-2 cells.

**Fig. 3:**
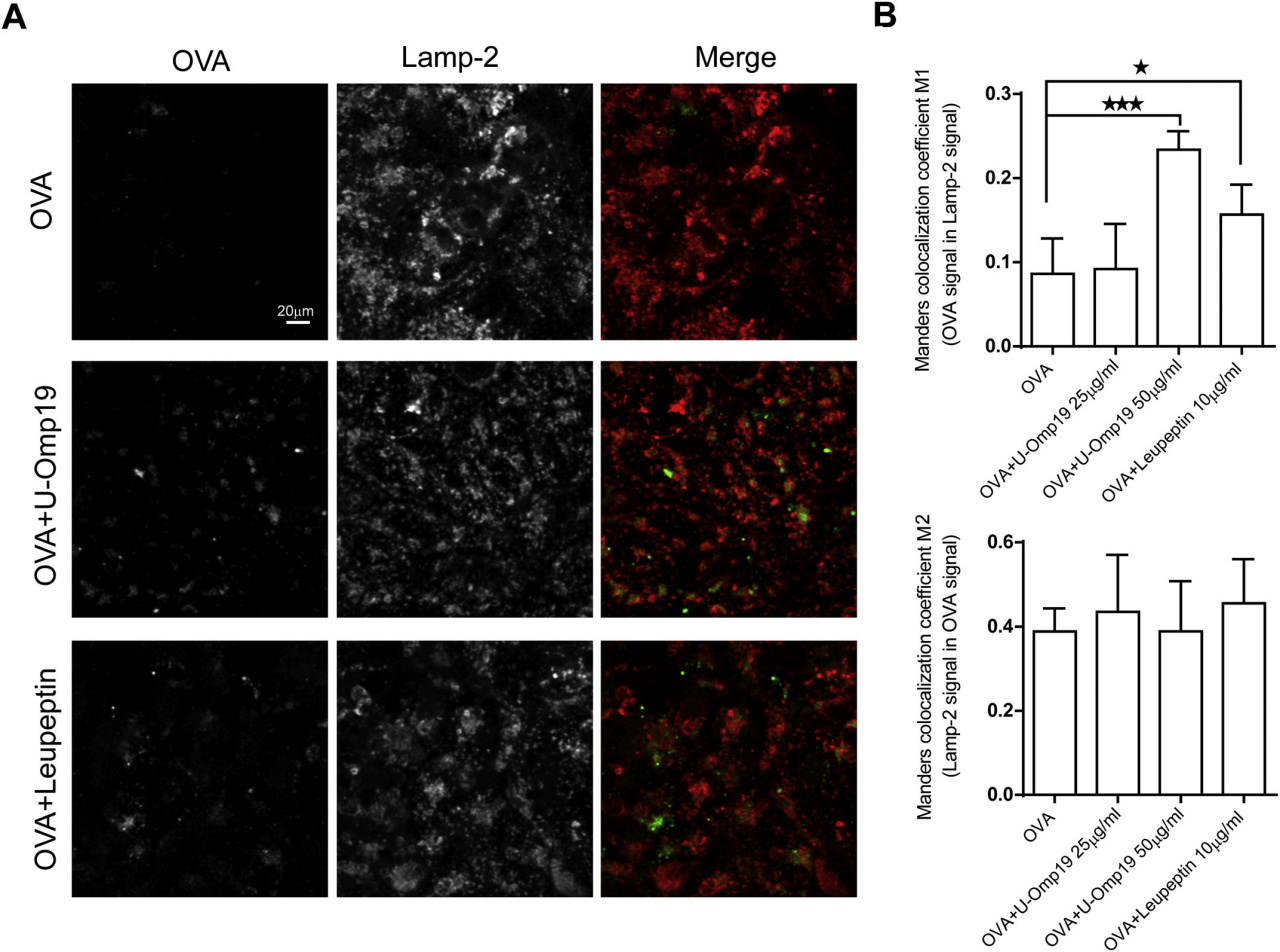
U-Omp19 co-delivery induces antigen accumulation at enterocytes lysosomes. Confocal scanning microscopy analysis of Caco-2 cells treated with OVA–Alexa Fluor 647 (25 μg/ml) alone or plus U-Omp19 (25 or 50 μg/ml) and Leupeptin (10 μg/ml). After 3 h of incubation cells were fixed, permeabilized, and stained with mAb anti–human Lamp-2. Anti-mouse IgG coupled to Alexa Fluor 546 (red) was used as secondary Ab. Images are representative of most cells examined by confocal microscopy. The merge between Lamp-2/OVA is shown. Quantification of colocalization OVA/Lamp-2 (M1) and Lamp-2/OVA (M2) was analyzed by Manders overlap coefficient. Data are means of Manders coefficients ± SEM present in 4–5 images of each condition. Scale bars 20 μm. * *p* < 0.05; ***p < 0.001 *vs* OVA. ANOVA followed by Bonferroni multiple comparison test. (For interpretation of the references to colour in this figure legend, the reader is referred to the web version of this article.)

Inhibition of lysosomal proteases by U-Omp19 could lead to accumulation of the Ag inside lysosomes of epithelial cells as it has been reported in our previous studies using DCs [8]. Therefore, intracellular Ag amount inside Caco-2 cell lysosomes after incubation with U-Omp19 was studied by confocal microscopy. Caco-2 cells monolayers were pulsed with OVA Alexa Fluor 647 (green) alone, with U-Omp19 or leupeptin for 3 h and then labeled with the endolysosomal associated marker Lamp-2 (red). A significant increase in Manders colocalization coefficient M1 between OVA and Lamp-2 signal after U-Omp19 (100 μg/ml) or Leupeptin treatment of the cells was observed (Fig. 3A–B) while there were no differences in M2 Manders coefficient (Lamp-2 signal in OVA signal) indicating that the Ag is reaching Lamp-2 positive compartments in all cases but is only being accumulated in presence of protease inhibitors. These results indicate that U-Omp19 inhibition of intracellular cysteine proteases promote accumulation of Ag inside lysosomes of Caco-2 cells and does not change intracellular fate of the co-administered Ag.

### 3.3 U-Omp19 facilitates the transcellular passage of Ag through epithelial cell monolayers

To reach lamina propria the Ag should bypass epithelial cell barrier at the mucosal surface. Thus, we investigated the effect of U-Omp19 on Ag transcytosis through Caco-2-polarized monolayers using Transwell filters. Measurements of TEER were performed during the cell culture to determine the formation of the epithelial barrier and then during the experiment to evaluate cell monolayer integrity. Of note, U-Omp19 addition did not induce any significant change in TEER values at any time (Supplementary Fig. 2).

**Fig. 4:**
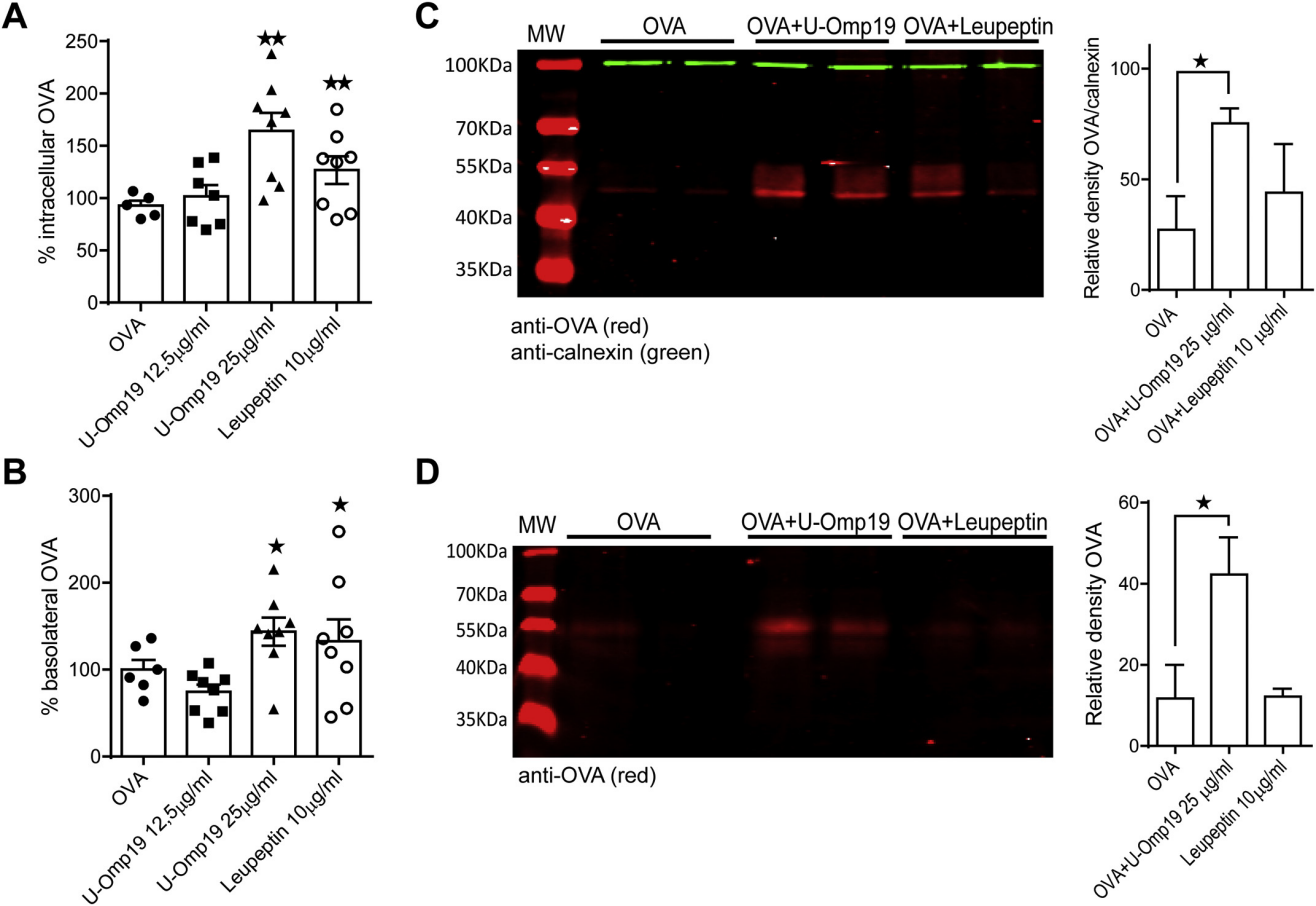
U-Omp19 facilitates Ag transport through epithelial cell monolayers. Transwell plates cultured Caco-2 cell monolayers were incubated with OVA-Alexa Fluor 647 alone (0.5 mg/ml) or in presence of U-Omp19 or Leupeptin during 3 h. Then, fluorescence in cell lysates (A) and basolateral medium (B) were measured in a microplate fluorescence reader. Result were pooled from 3 different experiments and data are means of percentage of OVA ± SEM. *p < 0.05; **p < 0.01 *vs* OVA. ANOVA followed by Bonferroni multiple comparison test. Western blot analysis and quantitative densitometry of OVA signal was performed in precipitated proteins of cell lysates (C) and basolateral medium (D). Calnexin was used as internal loading control in the case of cell lysates. Data are presented as means of relative density of OVA ± SD. *p < 0.05 *vs* OVA. ANOVA followed by Bonferroni multiple comparison test.

Caco-2 cell monolayers in Transwell filters were incubated at the apical side with OVA-AF647 alone, plus U-Omp19 or plus Leupeptin during 3 h. Medium from basal chamber was collected every hour for 3 h and the presence of OVA Alexa Fluor 647 was evaluated by fluorescence measurement. As shown in Fig. 4A, the amount of intracellular OVA was higher when cells were incubated with OVA plus U-Omp19 (25 μg/ml) or plus leupeptin. Then, emission of fluorescence was determined in the medium from lower chamber. Incubation with protease inhibitors U-Omp19 and leupeptin increased the transcytosis of OVA to the basolateral medium (Fig. 4B). To determine the proportion of intact protein (intracellular or transported) after incubation of enterocytes with U-Omp19 Western blots against OVA were performed in cell lysates and basolateral medium. Densitometry analysis revealed a significant increase of OVA in lysates and basolateral mediums from Caco-2 cells that were co-incubated with OVA+U-Omp19 in comparison with those incubated with OVA alone (Fig. 4C and D).

These results together indicate that U-Omp19, when co-delivered with an Ag, protects the Ag from digestion inside enterocytes allowing it to be transported across the intestinal epithelial barrier and then reach the lamina propria in a less digested manner.

### 3.4 U-Omp19 increase Ag internalization by DCs after its *in vivo* co-administration

**Fig. 5:**
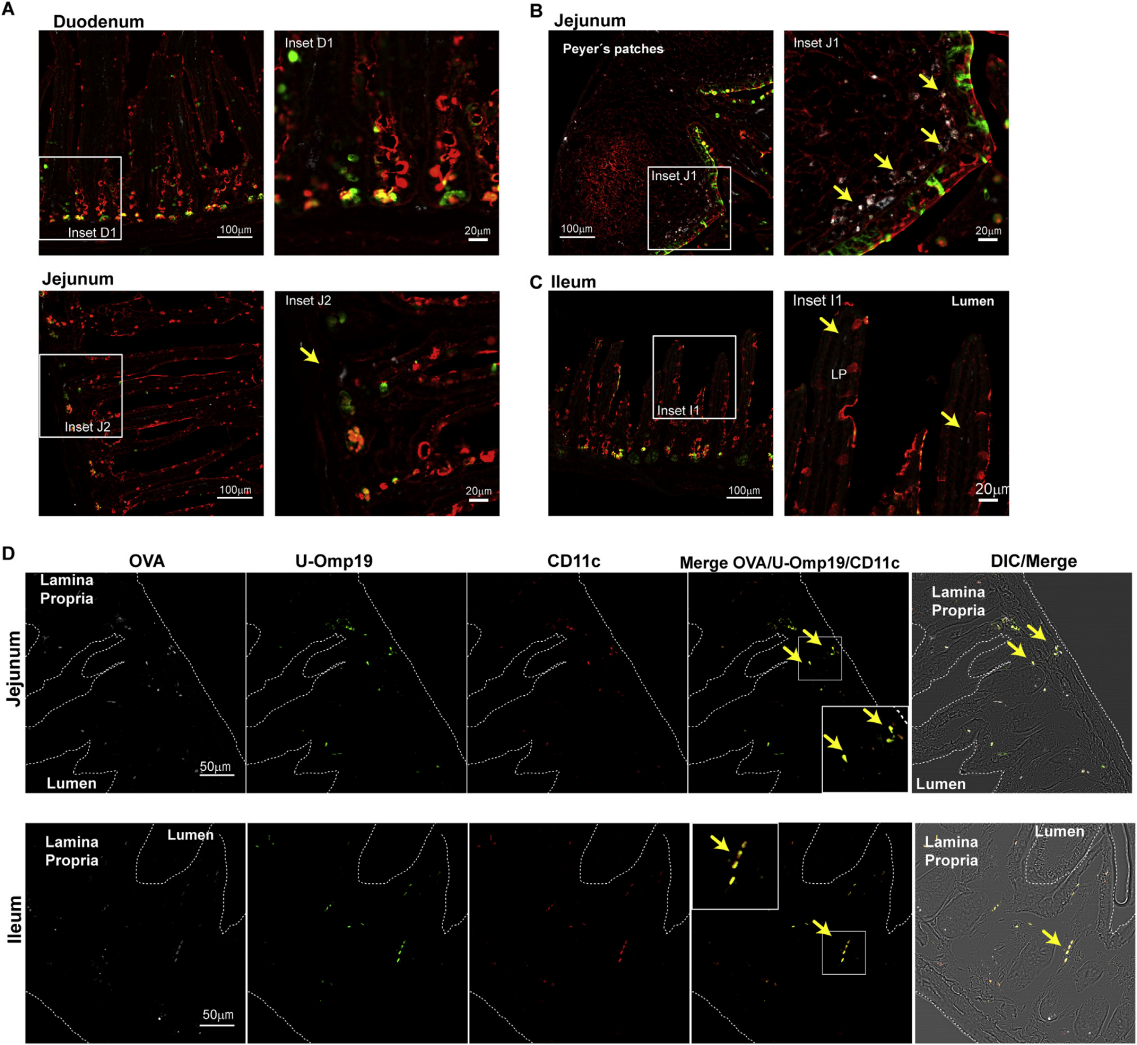
Ag fate after oral co-delivery with U-Omp19 in mice intestine sections. BALB/c mice were orally co-delivered with OVA-Alexa Fluor 647 (white) + U-Omp19 and 2 h later intestines were excised. Intestine cryosections were stained with UEA (green) and WGA staining (red) for epithelial cells. Yellow arrows indicate OVA-Alexa Fluor 647 (white) at duodenum (A), jejunum (B) and ileum (C). Images are representative of two independent experiments. Scale bars 100 μm (left panels, 10× objective) or 20 μm (inset panels). D. Jejunum and Ileum sections were labeled with rabbit anti-U-Omp19 polyclonal Ab or mouse anti-CD11c mAb as primary antibodies and then anti-rabbit Alexa Fluor 488 (green) or anti-mouse Alexa Fluor 546 (red) secondary antibodies. Images shown OVA-AF647 (white), U-Omp19 (green) or CD11c (red) signal separately and merge of 3 signals and DIC (right panels). Scale bars 50 μm. (For interpretation of the references to colour in this figure legend, the reader is referred to the web version of this article.)

To analyze the internalization of co-administered Ag *in vivo*, mice were orally administered with OVA-Alexa Fluor 647 alone or plus U-Omp19. After 2 h different intestinal sections (duodenum, jejunum and ileum) were excised and fixed cryosections were analyzed by immunofluorescence. At this time point, fluorescent Ag was found at the different intestine sections in all mice (Fig. 5 and Supplementary Fig. 3). After co-administration of U-Omp19, OVA was present mostly at the lamina propria of ileum and jejunum sections (Fig. 5B–C, Inset I1, J1 and J2) while few fluorescent OVA were found at duodenum sections (Fig. 5A, Inset D1). In jejunum sections OVA was found in structures in the lamina propria of the villous epithelium (Fig. 5B, Inset J2) as well as in Peyer's patches in the subepithelial dome next to the M cells rich follicle associated epithelium (FAE) (Fig. 5B, Inset J1).

Next and to evaluate the Ag and adjuvant fate at intestinal tissues, the different intestine sections were labeled with anti-CD11c mAb (red) and anti-U-Omp19 polyclonal Ab (green). As shown in Fig. 5D we found that OVA and U-Omp19 colocalized at CD11c^+^ cells (yellow arrows in merge) at jejunum and ileum sections after oral administration of OVA AF647 plus U-Omp19 in mice.

These observations support our previous *in vitro* results confirming that OVA is being efficiently transported through the intestinal epithelial barrier to DCs in the lamina propria *in vivo* after co-delivery with U-Omp19.

### 3.5 CD103^+^ CD8α^+^ DC population is increased at Peyer's patches after U-Omp19 oral co-administration

**Fig. 6:**
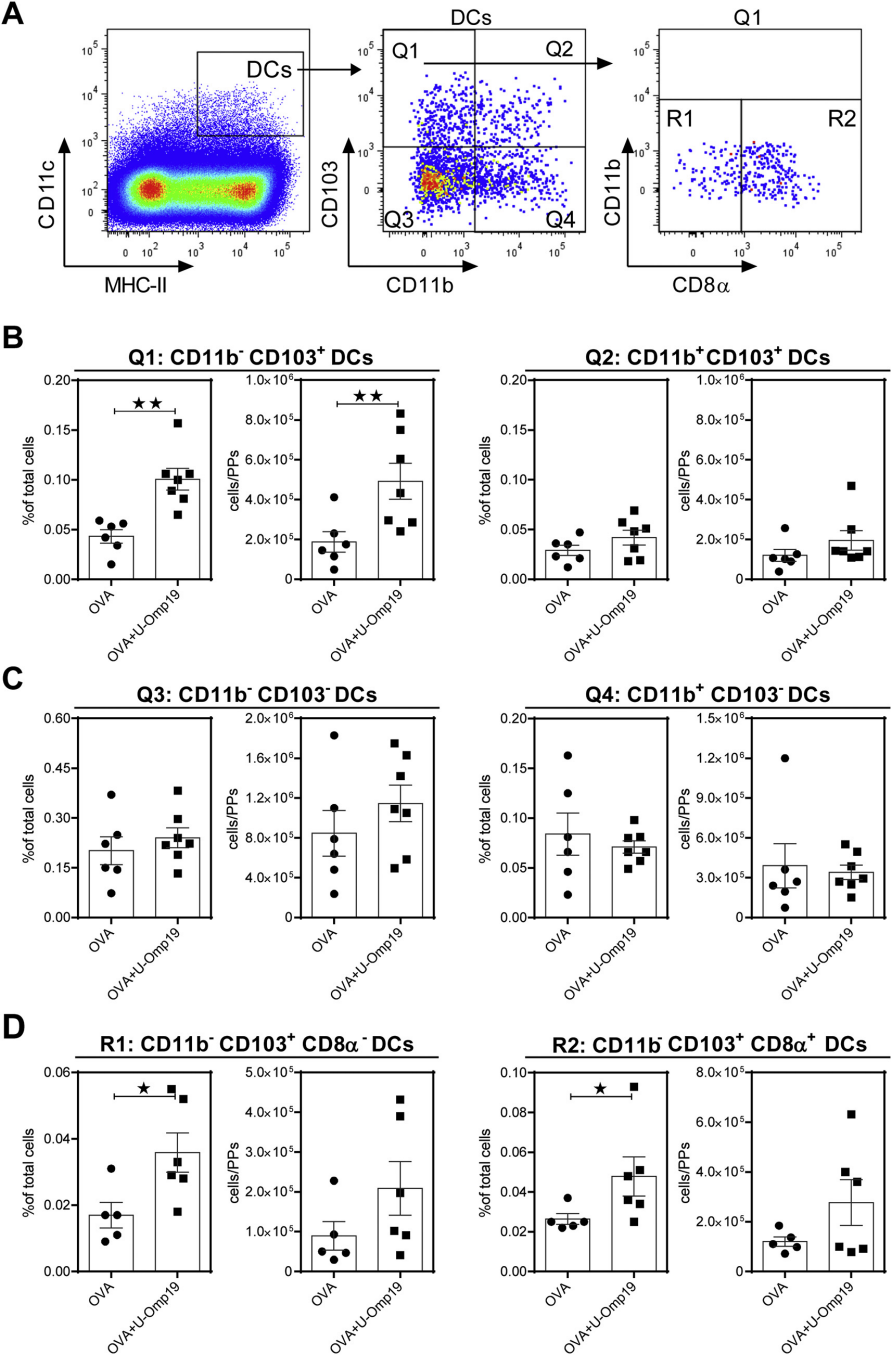
U-Omp19 increases dendritic cells subsets at Peyer's patches after oral delivery. BALB/c mice were orally administered with OVA alone or plus U-Omp19 and 18 h later DC populations were analyzed in Peyer's patches. Flow cytometry analysis of different DC populations were performed using anti-CD11c, anti-MHCII, anti-CD11b, anti-CD103, and anti-CD8α antibodies. Gating strategy is shown in A. Results are presented as percentage of total cells and in mean numbers of cells obtained for each mouse ± SEM and are representative of 3 different experiments. *p < 0.05, **p < 0.01 *vs.* OVA group. Mann Whitney *U* test.

After bypassing the epithelial barrier, the Ag should be captured by DCs that can present and activate naïve T lymphocytes to induce adaptive immune responses or on the contrary to induce immune tolerance. Besides, intestinal DCs comprise a highly heterogeneous population and each subset has diverse development and function [15]. In our previous work it has been demonstrated that oral administration of U-Omp19 induces CD11c^+^ CD8α^+^ DC subtype recruitment and activation at mucosal inductive sites and increases the amount of Ag within monocytes and DCs at Peyer's patches (PPs) and mesenteric lymph nodes (MLNs) [3]. Most recent studies have divided intestinal DCs on the basis of CD103 and CD11b expression [16,17]. Thus, further characterization of DC subtypes recruited after OVA plus U-Omp19 oral co-delivery was performed incorporating these markers. As shown in Fig. 6A–C at 18 h post oral administration the main DC population found at PPs was CD103^+^ CD11b^−^ CD11c^+^ MHCII^+^. Regarding CD8α expression, the percentage of both populations CD8α^+^ CD11b^−^ CD103^+^ CD11c^+^ MHCII^+^ and CD8α^−^ CD11b^−^ CD103^+^ CD11c^+^ MHCII^+^ was significantly increased at PPs in OVA+U-Omp19 immunized animals compared with the administration of OVA alone (Fig. 6D).

These results indicate that U-Omp19 induces the recruitment of intestinal CD103^+^ CD11b^−^ DCs at mucosal lymphoid tissues. These cells are involved in the induction of CCR9 and α4β7 expression on T cells and play a dominant role in cross-presenting intestinal epithelium derived Ags to CD8^+^ T cells in the steady state, required for optimal CD8^+^ T cell responses to orally administered protein antigens [15,17].

**Fig. 7:**
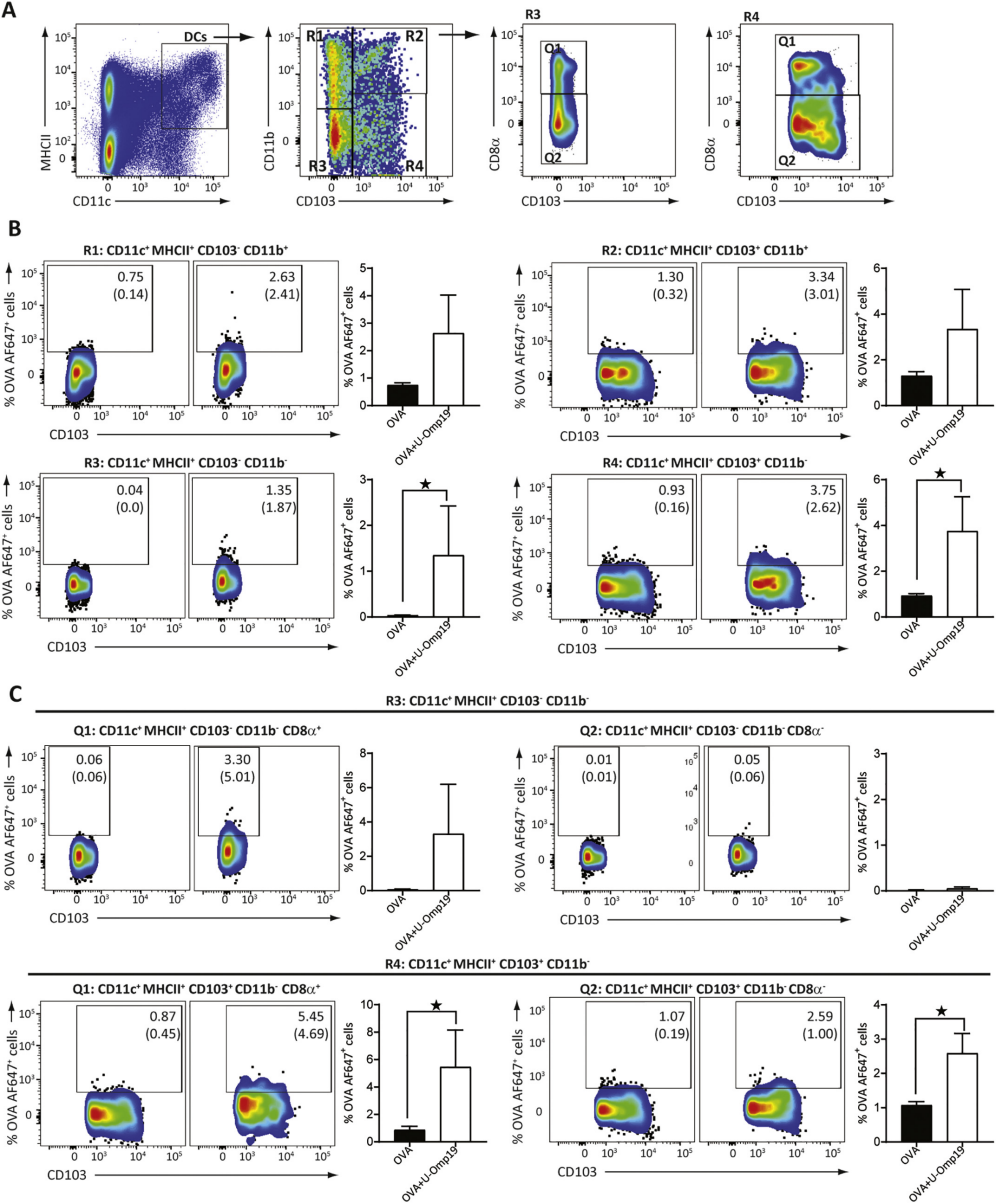
U-Omp19 in the oral vaccine formulation increases the frequency of mucosal DCs bearing the co-delivered Ag. BALB/c mice were orally administered with OVA Alexa Fluor 647 alone or plus U-Omp19 and 4 h later DC populations were analyzed in MLNs. Flow cytometry analysis of different DC populations were performed using anti-CD11c, anti-MHCII, anti-CD11b, anti-CD103, and anti-CD8α antibodies. Gating strategy is shown in A. Results are presented as percentage of OVA-AF647^+^ cells in the different populations of DCs (CD11c^+^ MHCII^+^) ± SEM. *p < 0.05 *vs.* OVA group. Mann Whitney U test.

Additionally, we evaluated by flow cytometry the phenotype of DCs containing OVA after its oral co-administration with or without U-Omp19 *in vivo*. As shown in Fig. 7A–B, there was a significant increase in the percentage OVA-AF647^+^ cells among CD103^+^ CD11b^−^ DCs and CD103^−^ CD11b^−^ DCs in the MLNs from mice receiving OVA-AF647 plus U-Omp19 in comparison with mice receiving OVA-AF647 alone. Furthermore, the percentage of both CD103^+^ CD11b^−^ CD8α^+^ DC and CD103^+^ CD11b^−^ CD8α^−^ DC subsets OVA-AF647^+^ was increased when U-Omp19 was co-administered (Fig. 7C). Thus, Ag oral administration with U-Omp19 increases the frequency of mucosal DCs bearing the co-delivered Ag.

### 3.6 U-Omp19 co-delivery with CTB increases Ag amount inside intestinal epithelial cells

Recombinant cholera toxin subunit B (rCTB) from *Vibrio cholerae* is the non-toxic portion of the cholera toxin and it has been assessed as Ag and adjuvant in human oral vaccine formulations [18]. CTB is used in the oral cholera vaccine Dukoral® which contains heat-killed whole cell *V. cholerae* and rCTB. This vaccine stimulates the production of both antibacterial and antitoxin antibodies, including secretory immunoglobulin A (S-IgA) produced locally in the intestines [19]. To evaluate U-Omp19's adjuvant properties with a well characterized Ag and licensed vaccine component, CTB was used as Ag co-administered orally with U-Omp19.

**Fig. 8:**
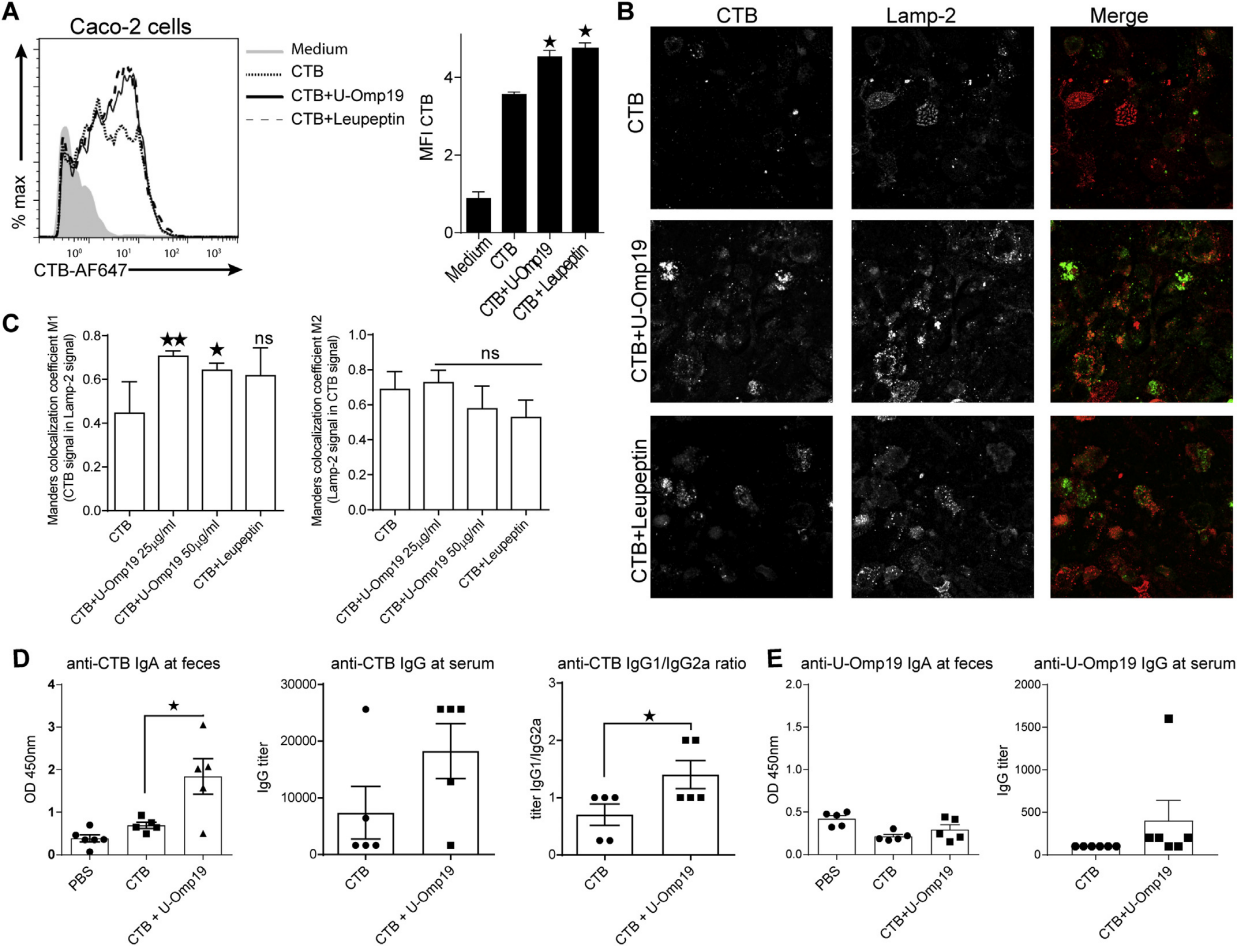
U-Omp19 increases CTB half-life and immunogenicity after oral co-delivery. A. Caco-2 epithelial cell line was incubated with CTB-Alexa Fluor 647 (10 μg/ml) alone or plus U-Omp19 (50 μg/ml) or Leupeptin (10 μg/ml) during 3 h and then MFI was determined by flow cytometry. Data are presented as histograms and bar graphs ± SEM. B. U-Omp19 co-delivery with CTB increases CTB accumulation inside the lysosome of intestinal epithelial cells. Confocal microscopy analysis of Caco-2 cells treated with CTB-Alexa Fluor 647 (10 μg/ml, green) alone or plus U-Omp19 (25 or 50 μg/ml) and Leupeptin (10 μg/ml). After 3 h of incubation cells were fixed, permeabilized, and stained with mAb anti–human Lamp-2. Anti-mouse IgG coupled to Alexa Fluor 546 (red) was used as secondary Ab. Images are representative of most cells examined by confocal microscopy. C. Quantification of colocalization was analyzed by Manders overlap coefficient. Data are means of Manders coefficients ± SEM present in 4–5 images of each condition. *p < 0.05; **p < 0.01 *vs* CTB alone. ANOVA followed by Bonferroni multiple comparison test. D-E. U-Omp19 induces specific antibody responses after oral co-delivery with CTB. BALB/c mice (*n* = 6/group) were orally immunized on days 1, 2, 3, 8, 9 and 10 with saline, CTB (10 μg) or CTB plus U-Omp19 (150 μg). Specific IgA and IgG levels anti-CTB and anti-U-Omp19 were evaluated in feces and sera by ELISA. Anti-CTB IgG1 and IgG2a levels were evaluated in sera. Data are means of OD 450 ± SEM for IgA responses and as IgG titers or titer ratio ± SEM for anti-CTB IgG, IgG1 or IgG2a. *p < 0.05 *vs* CTB group. Mann Whitney *U* test. (For interpretation of the references to colour in this figure legend, the reader is referred to the web version of this article.)

In agreement with the results obtained for OVA and Casein, the intracellular amount of CTB was significantly higher in Caco-2 cells incubated with CTB-Alexa Fluor 647 plus U-Omp19 in comparison with cells incubated with CTB alone (Fig. 8A). Moreover, confocal microscopy analysis on Caco-2 cells incubated with CTB-Alexa Fluor 647 in presence of U-Omp19 revealed a significant increment in the colocalization coefficient of CTB with Lamp-2 positive compartments (Fig. 8B–C). These results indicate that U-Omp19 co-delivery increases CTB amount inside enterocytes lysosomes as it has been demonstrated for other model proteins (OVA and Casein).

### 3.7 Production of anti-CTB mucosal IgA after co-immunization with U-Omp19

IgA is the most abundant produced antibody isotype, and a large portion of IgA is secreted into the intestinal lumen where it plays a major role as the first line of defense against adherence and invasion by enteric pathogens and neutralization of the toxins [1]. Measurement of IgA production at feces and IgG at serum was assessed after oral immunization of mice with CTB alone or plus U-Omp19. Two weeks after last immunization, U-Omp19 induced a significant increase in the production of anti-CTB IgA in feces in comparison to immunization with the Ag alone (Fig. 8D). Serum titers of anti-CTB IgG were higher but not statistically significant after mice immunization with CTB plus U-Omp19 group in comparison with the CTB alone group (Fig. 8D). Of note, immunization with CTB plus U-Omp19 modified the ratio of IgG1/IgG2a antibody titer promoting the predominance of IgG1 over IgG2a antibodies compared with immunization of CTB alone (Fig. 8D). Immunizations did not increase anti-U-Omp19 antibodies at mice feces or serum (Fig. 8E).

These results indicate that co-delivery of U-Omp19 by oral route induces the production of mucosal specific IgA, this response is key to control infections by preventing the attachment of pathogens or toxins to mucosal surfaces. Also, U-Omp19 regulates the Ag-specific IgG response changing the elicited antibody isotype profile.

Altogether our results demonstrate that U-Omp19 co-delivery protects Ag digestion inside enterocytes facilitating Ag transport across the intestinal epithelial barrier, increasing the amount of Ag that reaches the inductive sites for the induction of appropriate immune responses.

## 4 Discussion

The study of capture and processing of Ags by intestinal cells is very important for the development of new oral administration systems and vaccines. Ag digestion at the intestinal lumen and epithelium participates significantly in the degradation of epitopes which could give rise to specific immune responses. Thus, after bypassing luminal digestion by pancreatic and brush border proteases, other important feature of oral vaccine delivery systems should be to resist degradation during transcytosis through intestinal epithelial cells. A reduced epithelial barrier permeability and the susceptibility of Ags to proteolysis within the enterocytes, can affect the passage of Ag and the adjuvant through the intestinal epithelium, leading to a decrease in the uptake and presentation of Ags by lamina propria DCs and consequently lower immunogenicity of the formulation.

Brush-border membranes of epithelial cells are the second major enzymatic barrier in the GI tract, which can degrade both proteins and peptides. Then, after internalization, Ags are exposed to digestive enzymes inside lysosomes. In this work we have demonstrated that U-Omp19 has inhibitory activity on murine brush-border proteases from enterocytes. At the villous epithelium enterocytes have a key role in Ag digestion; small amounts of intact proteins are endocytosed by enterocytes, being subjected afterwards to cleavage within the lysosomal compartment. Studies revealed that large proteins taken up by enterocytes are released on their basal pole either as immunogenic peptides (~40%) or fully degraded into amino acids (~50%), with only a minor fraction crossing the epithelium in their intact form (<10%) [20]. Large peptides or proteins released into the lamina propria might then be taken up by local APCs [21]. Therefore, inhibition of proteolytic activity within enterocytes would contribute to protect the Ag during its transport across the epithelial barrier. This concept is supported by evidence that demonstrates that M cells -specialized in Ag transport at the intestinal epithelial surface- possess fewer lysosomes, indicating that a low intracellular antigen degradation ensures delivery of intact Ags to APCs at the lamina propria [22]. In this work we have demonstrated that U-Omp19 inhibits lysosomal cathepsin L activity inside live Caco-2 cells and promotes accumulation of Ag inside lysosomes of Caco-2 cells while does not change the intracellular fate of the co-administered Ag. Therefore, both the inhibition of brush border proteases and then lysosomal proteases from epithelial cells may contribute to the increment in Ag bioavailability observed at PPs and MLNs when U-Omp19 is co-delivered orally [5].

Using non-protein Ags, we also demonstrated that U-Omp19 does not have an effect in Ag uptake by Caco-2 cells suggesting that Ag intracellular accumulation (OVA and CTB) observed by confocal microscopy and flow cytometry could be explained by U-Omp19's protease inhibitory capacity on enterocytes. Indeed, we demonstrated that preventing Ag proteolysis inside enterocytes by mixing the Ag with a protease inhibitor enhance the amount of Ag (OVA) that traverse the epithelial barrier. Besides, OVA was found at the intestinal tissue both at the basolateral side of villous epithelium and inside Peyer's patches after its oral co-administration with U-Omp19. Even more, the Ag (OVA) and the adjuvant were found at CD11c positive cells in jejunum and ileum tissue sections after their oral co-administration indicating that the Ag is being transported across the intestinal epithelial barrier and reaches mucosal DCs *in vivo*.

In the case of adjuvants for oral vaccines, toxicity is an important issue to be addressed. Adjuvant reactogenicity is commonly linked to inflammatory properties [23]. We have previously shown that U-Omp19 does not produce any local reactions at the intestinal tissue after oral delivery in mice [7]. In concordance, U-Omp19 does not induce the production of the inflammatory cytokine IL-8 by epithelial Caco-2 cells after its incubation *in vitro* (data not shown).

It has been described that inhibition of matriptase – one of the key regulators in the formation and maintenance of epithelial barrier integrity- led to a decreased transepithelial electrical resistance (TEER) of the cell monolayer and to an enhanced paracellular transport of Ags [24]. In addition, disruption of intestinal epithelial barrier function has been reported for HIV protease inhibitors where gastrointestinal dysfunction represents a major adverse effect of this therapy [25]. The function of intestinal membrane permeation enhancers, commonly used in the oral delivery of peptides or proteins, has been also linked to toxicity issues [26]. Of note, formulations including U-Omp19 did not affect epithelial barrier permeability and integrity.

Polypeptide inhibitors have been used to great extent as auxiliary agents to overcome the enzymatic barrier of perorally administered therapeutic peptides and proteins [27] but were not included in vaccines until present. The efficacy of drug delivery systems containing the protease inhibitors aprotinin and bacitracin to improve the oral bioavailability of insulin has been demonstrated previously [28,29]. Although these protease inhibitors have a strong inhibitory potential against gastrointestinal proteases, they lack immunostimulatory properties. This is very important in the case of drug delivery where the induction of immune responses against the drug could cause a severe adverse effect. In contrast, mucosal delivery strategies of vaccine formulations should ensure not only overcome the harsh gastrointestinal environment but also an appropriate stimulation of immune system to avoid tolerance and achieve an effective protection.

We found that a specific DC population was increased at PPs after oral delivery of U-Omp19. This population expresses CD103 and CD8α marker but not CD11b. CD103^+^ DCs within PPs are better at inducing B-cells to become IgA-producing plasma cells when compared to their systemic counterparts [30]. Consistent with this idea, transport of Ags through goblet cell-associated passages were found to preferentially deliver antigen to CD103^+^ lamina propria DCs. Moreover, we demonstrated that Ag delivery in presence of U-Omp19 increased the percentage of CD11c^+^ MHCII^+^ CD11b^−^ CD103^+^ DCs containing Ag at MLNs at short times suggesting that this DC subset could be implicated in the type of immune response induced by U-Omp19. In mice, CD103^+^ LP DCs can migrate to the MLN [31] to initiate immune responses. In addition, CD103^+^ LP DCs generate retinoic acid [32], which is essential to imprint the expression of the gut homing receptors CCR9 and α4β7 on lymphocytes. A recent study demonstrated that migratory CD103^+^ CD11b^−^ CD8a^+^ are the only DC subset that present Ag acquired from enterocytes to CD8^+^ T cells [33] and were associated with cross-presentation of intestinal epithelial cell-derived antigens; induction of CD8^+^ effector T cells and Th1 cell priming [34]. CD103^+^ CD11b^−^ CD8a^+^ DCs population was increased after administration of U-Omp19 which could contribute to the induction of local Ag-specific Th1 and T CD8^+^ immune responses in mice. Immunostimulatory properties turns this protease inhibitor U-Omp19 into a very attractive compound to be used as adjuvant in oral vaccine formulations. Even more, this technology can be used alone or in combination with other oral delivery systems to optimize the efficiency of subunit vaccine formulations.

There are various mechanisms of action described for oral adjuvants. Some act by targeting Ags to M cells at the mucosal intestinal tissues, this is the case of cathelicidin LL-37 or CTA1-DD [35]. However, M cells comprise 5–10% of the follicle-associated epithelium [36] and the design of targeting strategies is extremely difficult *in vivo* due to highly variable proportion and phenotype among species [37]. Others, such as toxin derived adjuvants, possess ADP-ribosylating activity together with binding capacity to GM1 [38,39]. The mechanism of action of an adjuvant is complex and multifactorial, with many factors operating simultaneously or at different steps after its delivery. U-Omp19 oral co-administration as adjuvant increases the Ag half-life, induces the recruitment and activation of antigen presenting cells *in vivo* promoting specific adaptive immune responses [7,9] by mechanisms that comprise i) inhibition of Ag degradation at different steps in the gut: inhibition of stomach and lumen intestinal proteases, inhibition of brush border and lysosomal proteases from epithelial cells in addition to ii) immune stimulatory properties: recruitment of CD103^+^ CD11b^−^ CD8α^+^ DCs subset at the intestinal mucosa.

Cholera toxin subunit B has been used as Ag and adjuvant in human licensed vaccines against cholera for >25 years. We used CTB as Ag formulated with U-Omp19 as adjuvant and we observed increased CTB half-life inside enterocytes and specific-IgA production in feces after co-administration in mice. It is known that CT subunits can undergo transcytosis across epithelial cells from the apical to the basolateral surface [40]. Therefore, U-Omp19 could be useful to increase Ag-specific immune responses or to potentiate the effect of well-known antigens or adjuvants of already licensed vaccine compounds.

To summarize, in the present work we demonstrate that U-Omp19 interacts with the epithelial barrier: reduces the susceptibility of antigens to proteolysis in enterocytes (brush border and intracellular proteases) but does not change the integrity of the epithelial barrier. By limiting degradation, U-Omp19 enhances the passage of intact Ag and adjuvant through the intestinal epithelium leading to an increase in the uptake and presentation of antigens by the DCs, consequently providing greater immunogenicity to the formulation containing U-Omp19. Taken together, these data describe a new mechanism of action of a mucosal adjuvant never described till now and support the use of this rationale/strategy to develop new delivery systems for oral vaccines.

## 5 Conclusions

We have previously demonstrated that U-Omp19 has protease inhibitor activity and inhibits main gastrointestinal proteases protecting co-delivered Ags from digestion thus increasing immune responses. In this work, we further characterize its mechanism of action and demonstrate that the bacterial protease inhibitor U-Omp19 can interact with the epithelial barrier: inhibiting the susceptibility of antigens to proteolysis in enterocytes (brush border and intracellular proteases) but does not change the integrity and permeability of the epithelial barrier. Oral co-administration of U-Omp19 with an Ag, increases Ag availability inside enterocytes facilitating its transcellular passage through the epithelial barrier. This leads to an increase in the uptake and presentation of antigens by DCs and consequently enhances the immunogenicity of the formulation containing U-Omp19.

## Funding

This work was supported by grants from the Bill and Melinda Gates Foundation through the Grand Challenges Explorations Initiative (OPP1060394 and OPP1119024); from the Agencia Nacional de Promoción Científica y Tecnológica (ANPCyT-Argentina): PICT 2013 No 1500, PICT 2016 No 1310 (to JC).

## Author contributions

LMC, GSR and JC designed the experiments. Funding acquisition was done by JC. LMC GSR, KAP and LB performed all experimental assays. FFG collaborate with microscope acquisition and analysis. MCF collaborate in the experiments involving transwell filters. LMC and JC wrote the manuscript. All authors reviewed, commented, and approved the manuscript.

## Conflict of interest

LMC, KAP and JC are inventors of a patent related to U-Omp19. This patent, presented by the authors' National Research Council, “Adjuvant for vaccines, vaccines that comprise it and uses,” presentation P20090104015, was filed on October 19, 2009 in the National Institute of Intellectual Property, Argentina. This patent was also filed on October 18, 2010 in the European Patent Office, Spain PCT/ES2010/070667. The filing of the patent did not have any role in experimental design, data collection and analysis, decision to publish, or preparation of this manuscript. The authors have no financial conflicts of interest.

## Supplementary data

Supplementary material Image 1
