# Supplementary material for: Oral co-administration of a bacterial protease inhibitor in the vaccine formulation increases antigen delivery at the intestinal epithelial barrier
Source: J Control Release. 2019 Jan 10;293:158–71. doi: 10.1016/j.jconrel.2018.11.025 (PMC6329890; doi:10.1016/j.jconrel.2018.11.025)
Supplement: Supplementary file 1 — Supplementary material [file mmc1.doc]

**Supplementary Material**

**Supplementary 1.**

**
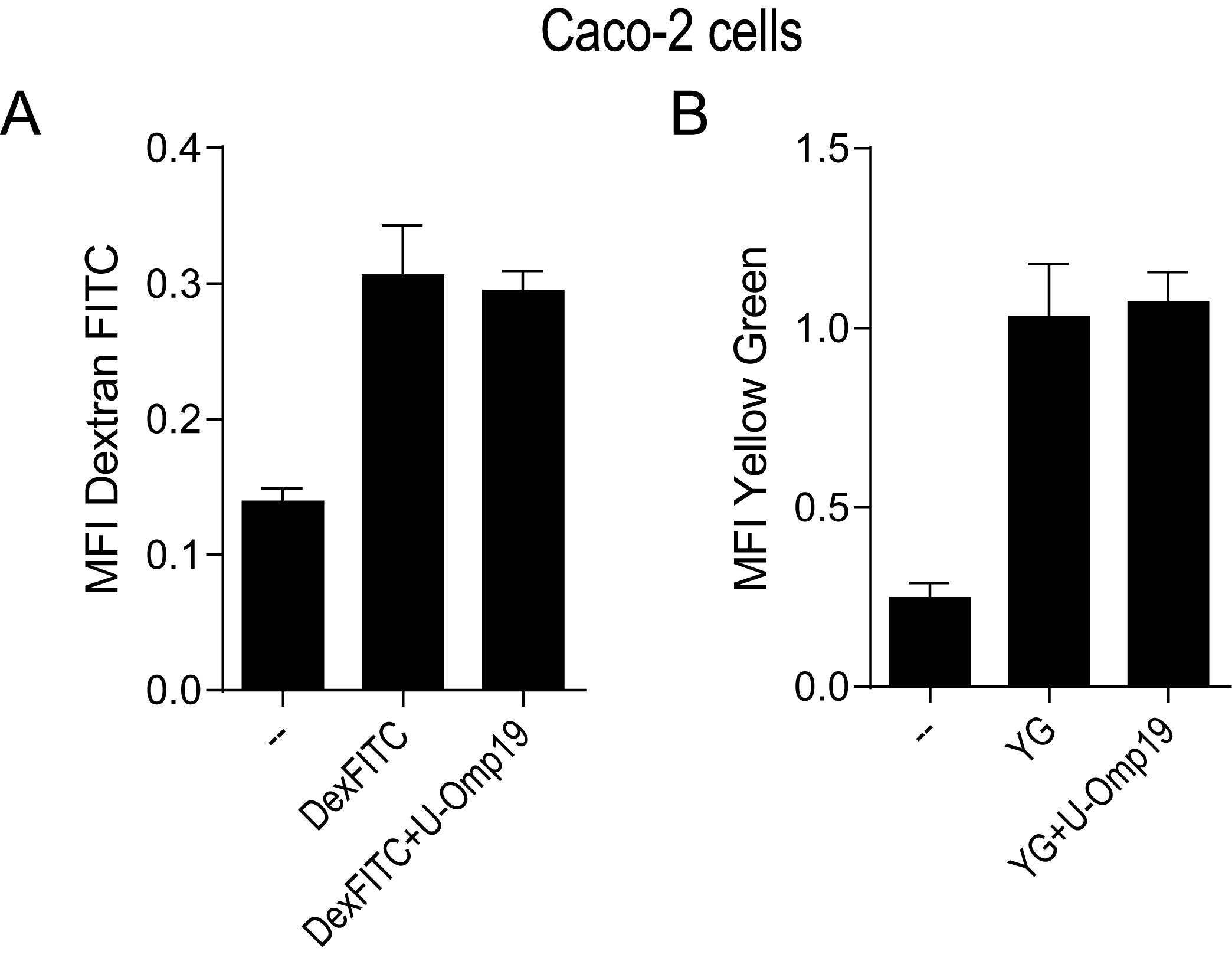
**

**Supplementary 1. U-Omp19 does not increase internalization of non-protein Ags.** Caco-2 epithelial cell line was incubated with (A) Dextran-FITC (Sigma, 70 KDa) or (B) Yellow green Particles (Polysciences Fluoresbrite microparticles, 1.024 um) alone or plus U-Omp19 during 3 h and then MFI was determined by flow cytometry. Data is presented as histograms and bar graphs ± SEM.


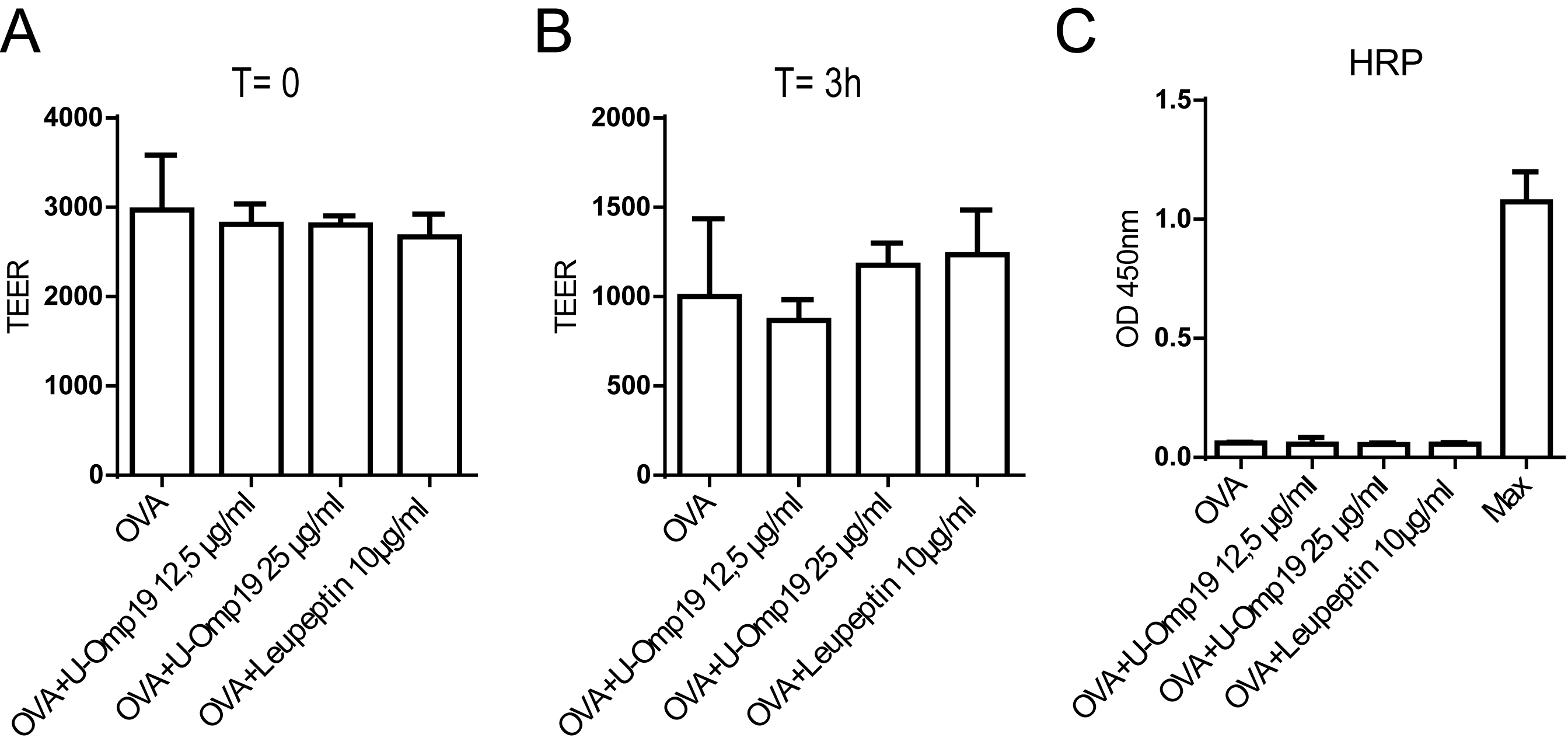
**Supplementary 2.**

**Supplementary 2. U-Omp19 formulations do not affect epithelial cell monolayer integrity.** Transepithelial electric resistance (Ω**.**cm) was measured in transwell filters containing Caco-2 cell monolayers before adding the Ag (**A**) and after 3 h of incubation with the formulations (**B**). **C**. Horseradish peroxidase (HRP, a nonspecific fluid-phase endocytosis marker) was used to monitor cell monolayer integrity after 3 h of incubation with the formulations. Data are means of OD 450 nm ± SED.

**Supplementary 3.**

**
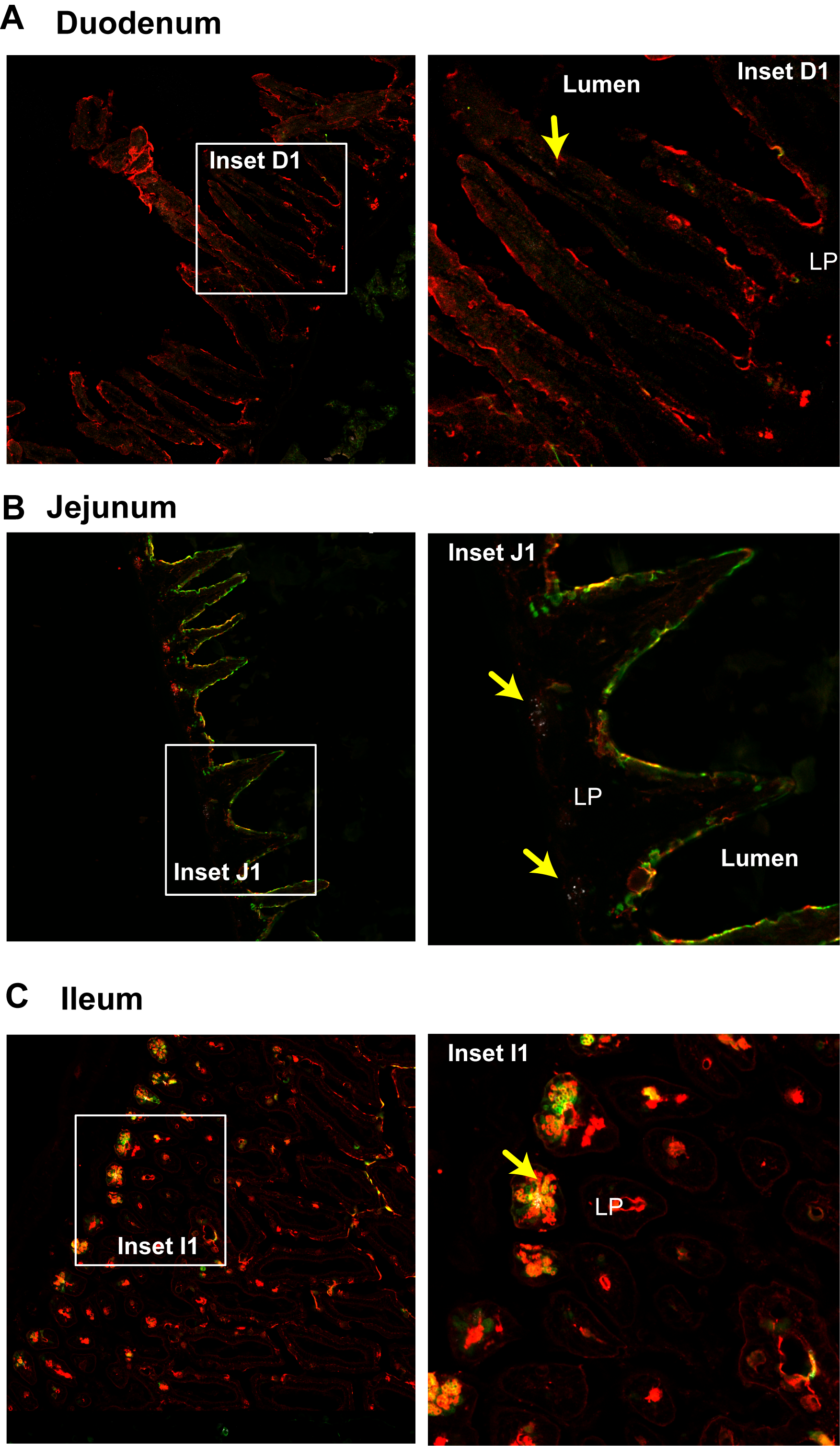
**

**Supplementary 3. Ag fate after oral delivery of OVA in mice intestine sections.** BALB/c mice were orally delivered with OVA-Alexa Fluor 647 and 2 h later intestines were excised. Intestine cryosections were staining with UEA (green) and WGA staining (red). Yellow arrows indicate OVA-Alexa Fluor 647 at duodenum (**A**), jejunum (**B**) and ileum (**C**). Images are representative of two independent experiments. Scale bars 100 µm (left panels, 10x objective) or 20 µm (inset panels).
